# Impact of preoperative indocyanine green injection on intraoperative decision-making and lymph node harvest in rectal cancer surgery

**DOI:** 10.3389/fsurg.2025.1683663

**Published:** 2025-09-26

**Authors:** Mario Pacilli, Giovanna Pavone, Ennio Lamanna, Arcangelo Picciariello, Michele De Fazio, Antonio Ambrosi, Nicola Tartaglia

**Affiliations:** 1 Department of Medical and Surgical Sciences, University of Foggia, Foggia, Italy; 2 Department of Experimental Medicine, University of Salento, Lecce, Italy; 3 Department of Precision and Regenerative Medicine and Ionian Area (DiMePRe-J), University of Bari Aldo Moro, Bari, Italy

**Keywords:** rectal cancer, minimally invasive surgery, indocyanine green, fluorescence, lymph node mapping

## Abstract

**Background:**

Real-time fluorescence-guided surgery using intraoperative indocyanine green (ICG) has gained increasing popularity in colorectal procedures. This study aims to assess the effectiveness of ICG fluorescence imaging in enhancing the intraoperative identification of lymph nodes and in reducing the rate of anastomotic leakage.

**Methods:**

A retrospective single-center study was conducted between September 2020 and December 2024 at a tertiary colorectal cancer surgery center. Patients with rectal cancer who underwent minimally invasive anterior rectal resection were included. They were divided into two groups: Group A received both preoperative peritumoral and intraoperative intravenous ICG injections, while Group B did not receive ICG. The intraoperative and short-term outcomes, including the number of harvested lymph nodes and the rate of anastomotic leakage, were compared between the groups.

**Results:**

A total of 40 patients (22 males) were included in the study. Operative time, hospital stay, intraoperative blood loss, and transfusion rates were similar between the two groups. Although the ICG group had a higher number of harvested lymph nodes (19 vs. 18), positive nodes, and lymph node ratio (LNR), these differences were not statistically significant on univariate analysis. Intraoperative changes were made in 30% of ICG cases to extend lymphadenectomy and in 25% to modify resection margins. Notably, no anastomotic leaks occurred in the ICG group (Group A), compared to a 10% leak rate in the control group (Group B). Linear regression analysis demonstrated that ICG use was significantly associated with increased lymph node yield (*β* = 3.65, *p* = 0.002), a higher number of positive nodes (*β* = 0.85, *p* = 0.028), and a greater LNR (*β* = 0.061, *p* = 0.034), indicating improved oncologic accuracy.

**Conclusions:**

This study demonstrates the feasibility and safety of using ICG fluorescence imaging in minimally invasive rectal cancer surgery. Its use could enhance lymph node mapping, support faster bowel recovery, and potentially reduce the risk of anastomotic leaks.

## Introduction

Minimally invasive surgery has become the standard approach for the treatment of rectal cancer, offering improved short-term outcomes and oncologic efficacy comparable to open procedures (1). However, challenges remain in achieving optimal lymphadenectomy and reducing postoperative complications, particularly anastomotic leakage, which continues to be a significant source of morbidity and mortality.

Indocyanine green (ICG) fluorescence imaging has emerged as a valuable tool in various surgical disciplines (2), enabling real-time visualization of vascular perfusion and lymphatic pathways. In colorectal surgery, ICG has shown promise in improving the assessment of bowel perfusion and facilitating more accurate lymph node mapping, both of which are critical for oncologic precision and surgical safety (3).

Despite growing interest in the application of ICG during rectal cancer surgery, the evidence supporting its routine use remains limited and heterogeneous (4).

When injected into peritumoral tissues, ICG migrates via the lymphatic pathways and accumulates in the regional lymph nodes, thereby enabling real-time mapping of tumor-specific lymphatic drainage (5). This method has shown promise in colorectal cancer surgery for improving the accuracy of lymphadenectomy.

Furthermore, the use of near-infrared fluorescence (NIRF) imaging with ICG enables real-time assessment of intestinal perfusion, contributing to improved anastomotic safety and a reduced risk of anastomotic leak (6).

Nowadays, robotic platforms such as the Da Vinci Xi system offer high-definition 3D visualization and enhanced tissue differentiation (7). When combined with ICG, these technologies may facilitate detailed intraoperative imaging of both vascular and lymphatic structures, without significantly extending operative time or disrupting workflow.

This study aims to evaluate the effectiveness of NIRF imaging with ICG in enhancing intraoperative identification of lymph nodes in patients undergoing minimally invasive surgery for rectal cancer.

## Methods

A retrospective study was conducted between September 2020 and December 2024 at the University Unit of General Surgery, Policlinico of Foggia. Patients diagnosed with rectal adenocarcinoma who underwent minimally invasive anterior rectal resection—either laparoscopic or robotic—were included.

Inclusion criteria comprised adults aged 18–80 years with histologically confirmed non-metastatic (M0) primary rectal cancer. Exclusion criteria included emergency procedures, pregnancy or breastfeeding, and known hypersensitivity to ICG. All participants provided written informed consent, and the study was conducted in accordance with the Declaration of Helsinki and Good Clinical Practice guidelines.

**Figure 1:**
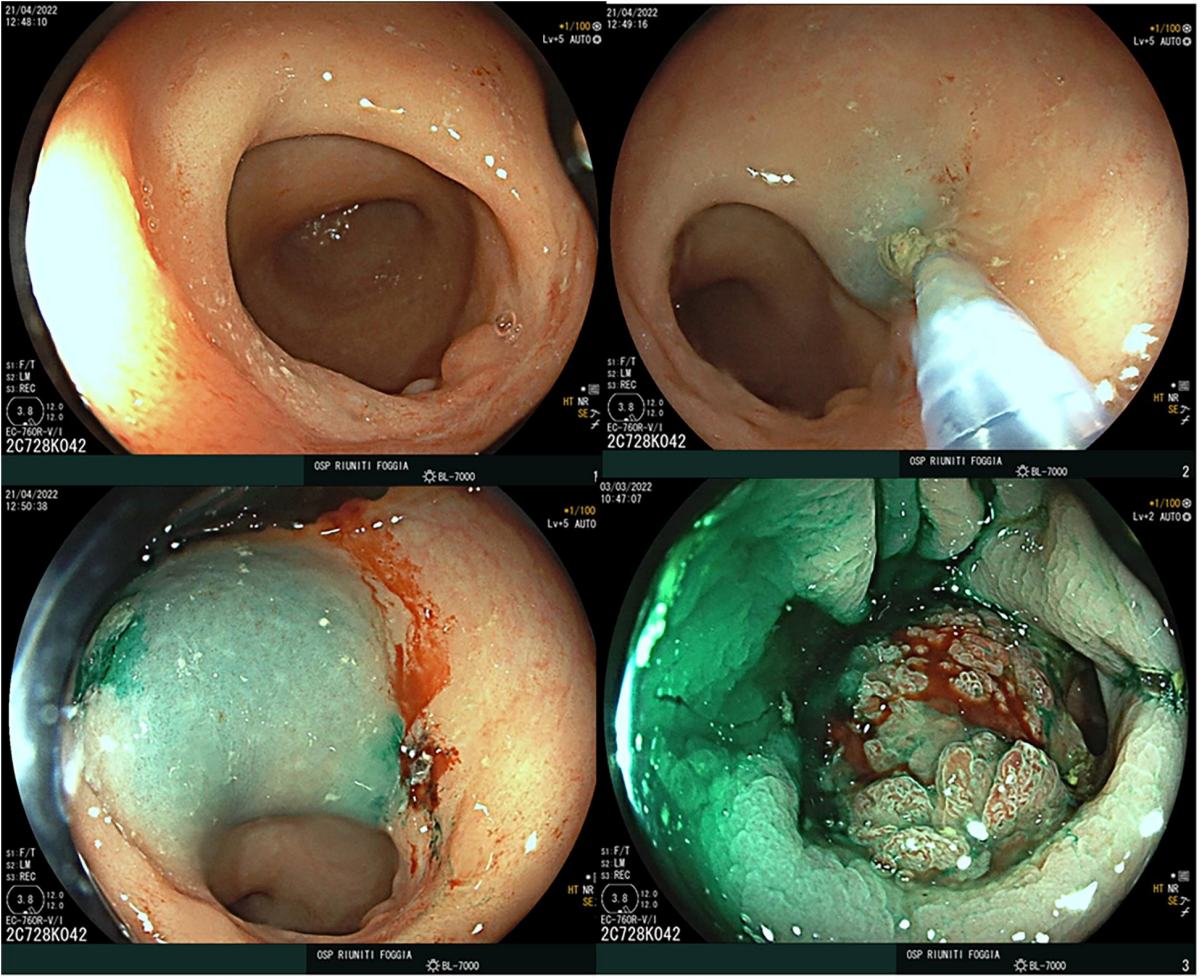
Endoscopic peritumoral injection of ICG.

Patients were divided into two groups:

- **Group A** underwent ICG-guided surgery, which included a preoperative endoscopic peritumoral injection of 3 mg of ICG (0.5 mg/mL dilution) into the four quadrants surrounding the tumor, administered 12–16 h before surgery (Figure 1), as well as intraoperative intravenous ICG administration.
- **Group B** underwent standard surgery without the use of ICG.

Patient allocation to Group A (ICG-guided) and Group B (non-ICG) was balanced throughout the study period, with both groups including patients from the early and later phases. All procedures were performed by a dedicated colorectal surgical team, with comparable expertise and experience throughout the study, ensuring consistency in surgical technique and perioperative management.

Preoperative staging for all patients included total colonoscopy with biopsy, chest and abdominal CT scans, pelvic MRI, and endoscopic ultrasound. For patients who underwent neoadjuvant chemoradiotherapy (CRT), restaging was performed following completion of treatment. Intraoperatively, near-infrared fluorescence imaging was employed in Group A to visualize lymphatic drainage pathways and to assess perfusion at the anastomotic site following intravenous administration of ICG (Verdye®, 5 mg/mL).

**Figure 2:**
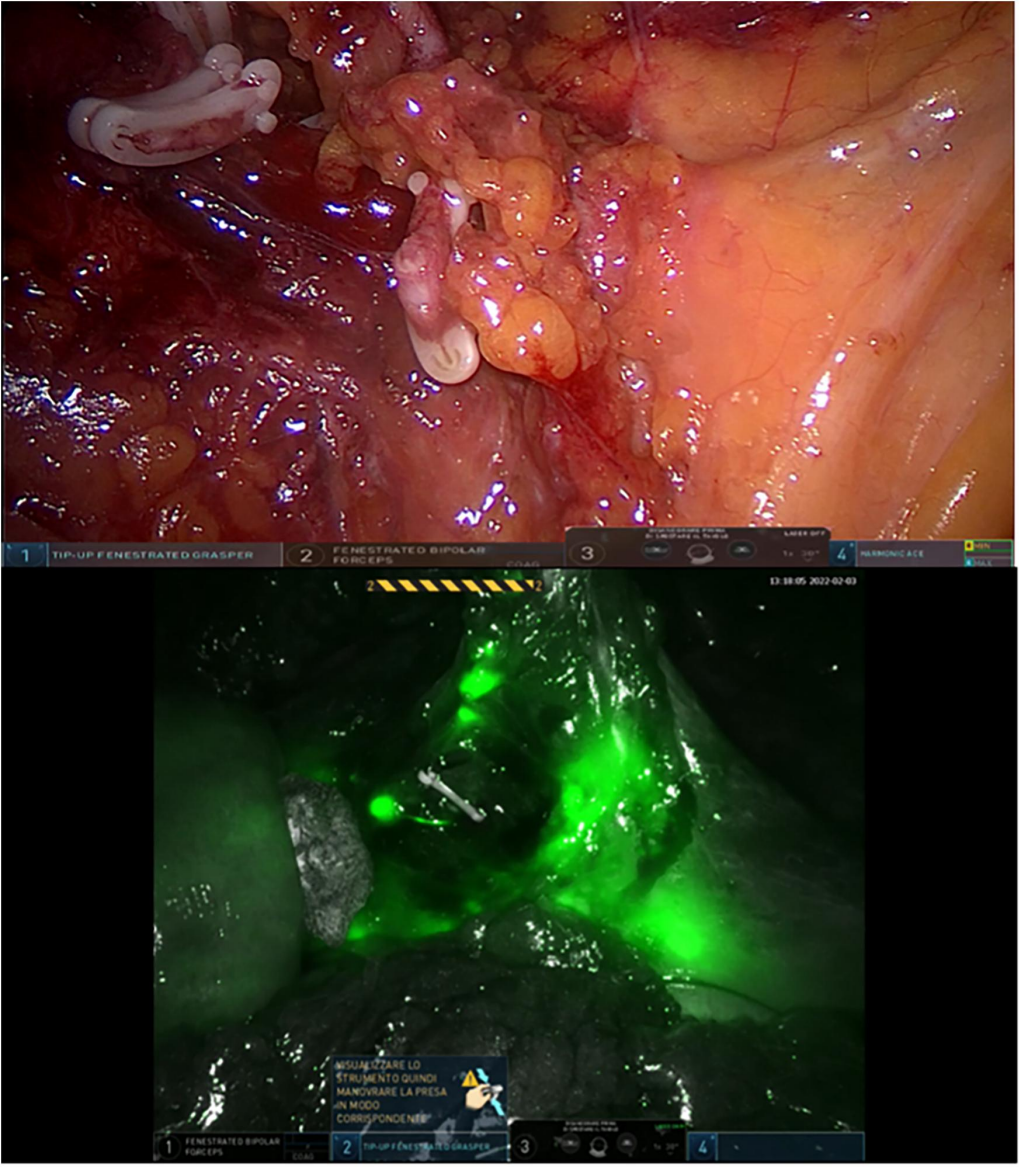
Pathological nodes, on the inferior mesenteric artery, detected with ICG.

**Figure 3:**
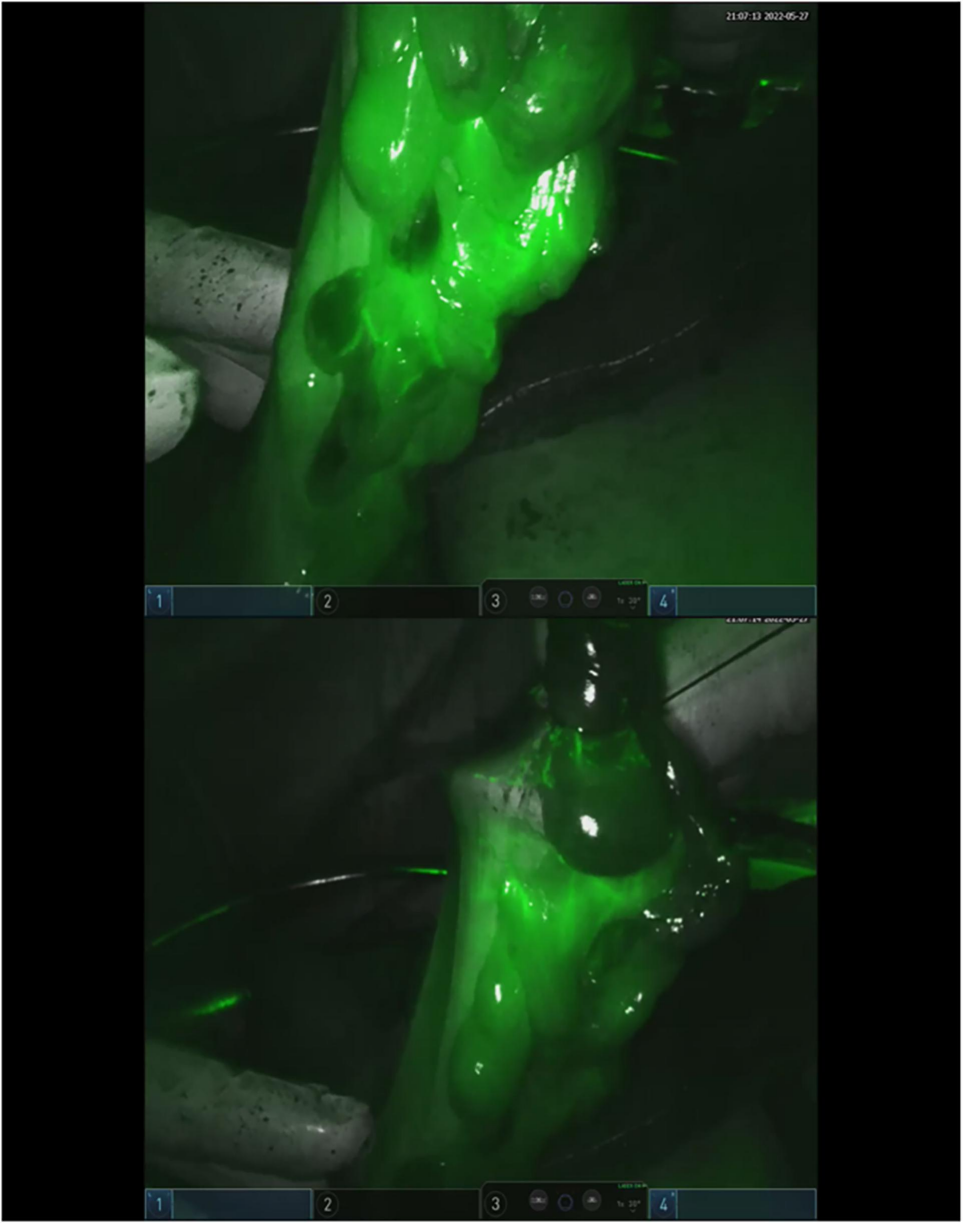
ICG on colonic stump to define the vascularization of the edges.

Clinical data, intraoperative observations, and postoperative outcomes were prospectively recorded in a dedicated Excel database. The primary outcomes assessed were the feasibility and safety of ICG use for lymph node mapping and perfusion evaluation. Comparative analyses between the two groups focused on total and positive lymph nodes retrieved, lymph node ratio (LNR), frequency of intraoperative modifications to lymphadenectomy, and incidence of clinically evident anastomotic leakage (Figures 2, 3).

## Statistical analysis

Statistical analyses were performed to evaluate differences in clinical, surgical, and pathological outcomes between the ICG group (Group A) and the non-ICG group (Group B). The distribution of continuous variables was assessed using the Shapiro–Wilk test. Continuous variables are expressed as median and interquartile range (IQR) and compared using the Wilcoxon signed-rank test. Categorical variables are reported as frequencies and percentages, and compared using the chi-square test.

To assess potential associations between the use of ICG and key surgical outcomes—including the number of harvested lymph nodes, number of positive nodes, and LNR—linear regression analyses were conducted. The level of statistical significance was set at *p* < 0.05. All analyses were performed using SPSS software, version 27.

## Results

Forty patients (22 males and 18 females) were enrolled in the study. Patients were equally distributed between the two groups, with no significant differences in baseline characteristics. The baseline characteristics of all patients are reported in Table 1.

**Table 1:** General data comparison between Group A and Group B.

General data	All patients	Group A	Group B	*p*-value
Age, years				
Median (IQR)	67.1 (11.8)	68.8 (10.8)	67.7 (12.2)	0.874^a^
Male/female	22/18	10/10	12/8	0.731^b^
BMI, kg/m^2^				
Median (IQR)	28.3 (10.5)	28.7 (10.3)	27.9 (10.5)	0.726^a^
Chronic disease (yes/no)	27/13 (67.5%–32.5%)	14/6 (70%–30%)	13/7 (65%–35%)	0.735^b^
Cardiovascular diseases	24/40 (60%)	13/20 (30%)	11/20 (55%)	0.518^b^
Pulmonary diseases	12/40 (30%)	6/20 (30%)	6/20 (30%)	–
Other	8/40 (20%)	4/20 (20%)	4/20 (20%)	–
ASA score 1–4	8 (20%), 19 (47.5%), 13 (32.5%)	3 (15%), 10 (50%), 7 (35%)	5 (25%), 9 (45%),6 (30%)	–
Tumor site				
Upper rectum	15 (37.5%)	7 (35%)	8 (40%)	0.731^b^
Middle	19 (47.5%)	9 (45%)	10 (50%)	0.751^b^
Lower	6 (15%)	4 (20%)	2 (10%)	0.784^b^
Neoadjuvant therapy, yes/no	18/22 (45%–55%)	10/20 (50%)	8/20 (40%)	0.525^b^
Clinical stage				
<T2N0	7/40 (17.5%)	3/20 (15%)	4/20 (20%)	0.677^b^
>T2N+	33/40 (82.5%)	17/20 (85%)	16/20 (80%)	0.678^b^

ASA, American Society of Anesthesiologists.

a Statistical analysis was performed using the Wilcoxon signed-rank test.

b Statistical analysis was performed using the chi-square test.

Table 2 shows a comparative analysis between intra- and postoperative data between Group A (ICG) and Group B (no ICG). The distribution of laparoscopic and robotic approaches was equal in both groups. The conversion rate to open surgery rate was 20% in Group A and 30% in Group B (*p* = 0.346). The median operative time was comparable between groups. Postoperative hospital stay was slightly shorter in Group A, but with no statistical significance (*p* = 0.128). Intraoperative blood transfusion was required in 15% of patients in Group A and in 30% in Group B (*p* = 0.451). Estimated blood loss was similar between the groups (*p* = 0.447). No 30-day mortality occurred in either group.

**Table 2:** Comparison between Group A and Group B.

Variable	All patients	Group A	Group B	*p*-value
Time of surgery (min)				
Median (IQR)	237 (21.6)	235 (17.2)	240.4 (29.5)	0.381^a^
Postoperative hospital stay (days)				
Median (IQR)	6 (1)	6 (0.75)	7 (1)	0.128^a^
Intraoperative blood transfusion	9/40	3/20 (15%)	6/20 (30%)	0.451^b^
Bleeding				
Median (IQR)	118 (73.75)	120 (33.5)	115 (28.62)	0.447^a^
Laparoscopic/robot	19/21	9/11	10/10	
Conversion to open surgery	10/40	4/20 (20%)	6/20 (30%)	0.346^b^
Gas passage (days)				
Median (IQR)	1.5 (1)	1 (1)	2 (2)	0.046
Bowel movement (days)				
Median (IQR)	1.5 (1)	1 (1)	1.5 (1)	0.298
Resumption of oral intake (days)				
Median (IQR)	1 (1)	1 (1)	2 (1)	0.061
Drainage duration (days)				
Median (IQR)	6 (1)	6 (0.75)	6.5 (1)	0.317
30-day mortality	–	0.00%	0.00%	–

a Statistical analysis was performed using the Wilcoxon signed-rank test.

b Statistical analysis was performed using the chi-square test.

The median number of harvested and positive lymph nodes was slightly higher in Group A (19) than that in Group B (18), although the difference was not statistically significant (*p* = 0.162). Notably, in 30% of cases in Group A, the extent of lymphadenectomy was adjusted intraoperatively based on ICG fluorescence guidance. No difference in LNR was found between the groups (*p* = 0.372). The quality of total mesorectal excision (TME) or partial mesorectal excision (PME) was optimal in 100% of patients in Group A, compared to 75% in Group B. In the remaining five cases from Group B, the quality of excision was classified as grade 2 according to the Quirke classification (8). Oncologic margins were also assessed in both groups. The circumferential resection margin (CRM) and distal resection margin were free of tumor involvement in all cases, with no significant differences observed between Group A and Group B.

Intraoperative adjustments to the colonic resection margin were made in 25% of cases in Group A, whereas no such adjustments were reported in Group B. No anastomotic leak occurred in Group A, whereas Group B experienced a 10% leak rate, which was treated conservatively Table 3).

**Table 3:** Comparison between Group A and Group B.

Variable	All patients	Group A	Group B	*p*-value
Harvested lymph nodes				
Median (IQR)	18.5 (4.26)	19 (5.75)	18 (4)	0.162
Positive lymph nodes				
Median (IQR)	2 (2)	2,5 (3)	2 (2)	0.141
Adjustment of the extent of lymphadenectomy	–	6/20 (30%)	–	–
LNR				
Median (IQR)	0.108 (0.114)	0.147 (0.153)	0.098 (0.089)	0.372
Quality of TME or PME	–	100%	75%	–
Adjustment of the colon resection	–	5/20 (25%)	–	–
Anastomotic leak	–	0%	2/20 (10%)	–

To assess the potential association between the use of ICG and surgical and pathological outcomes, a linear regression analysis was performed. The primary objective was to assess whether ICG administration influenced the number of harvested lymph nodes, the number of positive lymph nodes, and the LNR (Table 4).

**Table 4:** Linear regression analysis.

Outcome Variable	Coefficient (*β*)	95% CI	Standard error	*p*-value	*R*²
Harvested lymph nodes	3.65	1.45–5.85	1.06	0.002	0.26
Positive lymph nodes	0.85	0.10–1.60	0.36	0.028	0.13
LNR	0.061	0.005–0.117	0.027	0.034	0.12

Linear regression analyses demonstrated that ICG use was significantly associated with an increased number of harvested lymph nodes (*β* = 3.65, 95% CI: 1.45–5.85, *p* = 0.002), a higher number of positive nodes (*β* = 0.85, *p* = 0.028), and a greater LNR (*β* = 0.061, *p* = 0.034). These findings support the hypothesis that ICG-guided mapping may enhance nodal yield and detection (Table 4).

## Discussion

This study evaluated the impact of ICG with NIRF imaging on short-term surgical and oncologic outcomes in minimally invasive rectal cancer surgery. Our findings demonstrate that ICG use is associated with enhanced lymph node retrieval and more frequent intraoperative adaptations to both resection margins and lymphadenectomy.

Although the overall lymph node yield between groups was not statistically significant, linear regression analysis confirmed a significant association between ICG use and increased lymph node harvest. Furthermore, both the number of positive lymph nodes and the LNR were significantly higher in the ICG group, suggesting improved sensitivity for detecting N+ disease. This aligns with previous reports indicating that peritumoral ICG injection enables more accurate mapping of individual lymphatic drainage patterns, potentially revealing aberrant nodal basins that are not routinely dissected during standard oncologic resections (9, 10).

Importantly, 30% of patients in the ICG group required intraoperative modifications to the extent of lymphadenectomy based on fluorescence guidance, highlighting the dynamic utility of ICG imaging for tailoring surgery in real time. A study by Watanabe et al. (11) similarly observed that ICG mapping enabled more targeted lymphadenectomies without compromising resection margins or increasing operative times.

No anastomotic leaks occurred in Group A, compared to a 10% leak rate in the control group. Although the absolute numbers were small, the difference is clinically meaningful and mirrors the trend observed in larger prospective trials such as PILLAR II, where perfusion assessment using ICG reduced leak-related reinterventions (12). In our cohort, 25% of ICG cases required intraoperative modification of the resection margin, likely contributing to the improved anastomotic integrity.

The quality of TME/PME was also superior in the ICG group, with 100% achieving optimal dissection, compared to only 75% in the non-ICG group. The ability of ICG to enhance visual discrimination of vascular and lymphatic structures may support more precise dissection, particularly when combined with robotic platforms that offer stable, magnified visualization (13, 14).

Despite these encouraging outcomes, the study has some limitations. This was a retrospective and single-center study with potential selection bias. Although baseline characteristics were balanced, unmeasured confounders may have influenced surgical decision-making. The sample size was relatively small, and although statistical associations were demonstrated, larger multicenter studies are necessary to validate these findings. In addition, long-term oncologic outcomes such as disease-free and overall survival were not assessed.

In conclusion, the integration of ICG fluorescence imaging into minimally invasive rectal cancer surgery enhances intraoperative visualization, enabling more accurate lymph node mapping and safer anastomotic construction. While these results are promising, larger prospective studies are warranted to confirm the oncologic benefits and to standardize the use of ICG in routine colorectal practice.

## Data Availability

The raw data supporting the conclusions of this article will be made available by the authors, without undue reservation.
